# *Lactococcus lactis* KF140 Reduces Dietary Absorption of N^ε^ - (Carboxymethyl)lysine in Rats and Humans *via* β-Galactosidase Activity

**DOI:** 10.3389/fnut.2022.916262

**Published:** 2022-06-24

**Authors:** Ho-Young Park, Hye-Bin Lee, So-Young Lee, Mi-Jin Oh, Sang Keun Ha, Eunju Do, Hyun Hee L. Lee, Jinyoung Hur, Kwang-Won Lee, Mi-Hyun Nam, Myoung Gyu Park, Yoonsook Kim

**Affiliations:** ^1^Food Functionality Research Division, Korea Food Research Institute, Jeollabuk-do, South Korea; ^2^Clinical Trial Convergence Commercialization Team, Daegu Technopark, Daegu, South Korea; ^3^Department of Biotechnology, College of Life Sciences and Biotechnology, Korea University, Seoul, South Korea; ^4^Department of Ophthalmology, Sue Anschutz-Rodgers Eye Center, University of Colorado, Aurora, Aurora, CO, United States; ^5^MetaCenTherapeutics, Suwon, South Korea

**Keywords:** *Lactococcus lactis* (*L. lactis*), advanced glycation end product (AGE), N^ε^-(carboxymethyl)lysine (CML), β-galactosidase, probiotic

## Abstract

**Background and Aims:**

Excessive intake of advanced glycation end products (AGEs), which are formed in foods cooked at high temperatures for long periods of time, has negative health effects, such as inflammatory responses and oxidative stress. N^ε^-(Carboxymethyl)lysine (CML) is one of the major dietary AGEs. Given their generally recognized as safe status and probiotic functionalities, lactic acid bacteria may be ideal supplements for blocking intestinal absorption of food toxicants. However, the protective effects of lactic acid bacteria against dietary AGEs have not been fully elucidated.

**Materials and Methods:**

We investigated the effect of treatment with *Lactococcus lactis* KF140 (LL-KF140), which was isolated from kimchi, on the levels and toxicokinetics of CML. The CML reduction efficacies of the *Lactococcus lactis* KF140 (LL-KF140), which was isolated from kimchi, were conducted by *in vitro* test for reducing CML concentration of the casein-lactose reaction product (CLRP) and *in vivo* test for reducing serum CML level of LL-KF140 administered rats at 2.0 × 10^8^ CFU/kg for14 days. In addition, 12 volunteers consuming LL-KF140 at 2.0 × 10^9^ CFU/1.5 g for 26 days were determined blood CML concentration and compared with that before intake a Parmesan cheese.

**Results:**

Administration of LL-KF140 reduced serum CML levels and hepatic CML absorption in rats that were fed a CML-enriched product. In a human trial, the intake of LL-KF140 prevented increases in the serum levels of CML and alanine aminotransferase after consumption of a CML-rich cheese. LL-KF140 was determined to presence in feces through metagenome analysis. Furthermore, β-galactosidase, one of the *L. lactis*-produced enzymes, inhibited the absorption of CML and reduced the levels of this AGE, which suggests an indirect inhibitory effect of LL-KF140. This study is the first to demonstrate that an *L. lactis* strain and its related enzyme contribute to the reduction of dietary absorption of CML.

## Introduction

The advancement of food science has brought convenience to humans and has helped improve the storage, safety, functionality, and flavor of food. In particular, the heating process adds flavor to food and improves its storage properties. However, heating also generates undesired compounds, such as acrylamide, trans fat, and advanced glycation end products (AGEs), the continuous ingestion of which causes inflammation and chronic diseases (1–3). AGEs are a result of non-enzymatic reactions between carbonyl groups of reducing sugars and free amino groups in foods cooked at high temperatures or preserved for long periods (4). AGEs are also formed in the body during normal cellular metabolism (5). Elevation of endogenous or exogenous AGE levels is associated with inflammation, oxidative stress, reduced endothelial function, insulin resistance, and metabolic disorders (6). Important AGEs include pentosidine, *N*^ε^-(carboxymethyl)lysine (CML), and hydroimidazolone, among which CML is the most abundant and has been widely studied in foods (7).

Lactic acid bacteria (LAB), which are capable of fermenting lactose into lactate, have been reported to remove toxic substances, such as carcinogenic compounds and mycotoxins, in food applications (8, 9). Traditional lactic acid fermentation, one of the oldest practices in food preparation, may have high potential as an alternative and practical strategy for the mitigation of mycotoxins in foods. Some LAB strains bind mycotoxins to the cell wall or induce their enzymatic degradation, thus reducing mycotoxin contamination (9). In addition, successful applications of LAB have been reported in the removal of toxic heavy metals, including mercury and arsenic, and detoxification of acrylamides (10, 11). Considering their generally recognized as safe (GRAS) status and probiotic functionalities, LAB may be an ideal treatment to prevent the occurrence or reduce the levels of food toxicants *via* blocking their intestinal absorption. However, the protective effects of beneficial bacteria against dietary AGEs have not yet been fully elucidated.

The present study aimed to evaluate the effect of treatment with a *Lactococcus lactis* strain that was isolated from kimchi, a traditional Korean fermented food, on the levels and toxicokinetics of CML and investigate the direct mechanism by which probiotics can influence the detoxification of AGEs. In addition, the enzymatic role of *L. lactis* in CML reduction was evaluated and specific enzyme was suggested.

## Materials and Methods

### Isolation and Phylogenetic Analysis of Strain KF140 as Lactococcus lactis (LL-KF140)

LL-KF140 was isolated from kimchi and identified by 16S rRNA gene sequence analysis. The bacterial 16S rRNA gene was amplified by polymerase chain reaction using two universal primers, 8F (5’-AGAGTTTGATCCTGGCTCAG-3’) and 1492R (5’-CGGTTACCTTGTTACGACTT-3’). The EzTaxon-e server^1^ was used to identify the phylogenetic neighbors of LL-KF140 and to calculate the pairwise 16S rRNA gene sequence similarities (12). The 16S rRNA gene sequences of LL-KF140 (1,490 bp) and closely related type strains were aligned using CLUSTALW.^2^ Phylogenetic trees were constructed using the neighbor-joining algorithm (13) and maximum-likelihood algorithm using Molecular Evolutionary Genetics Analysis software version 6.06. The bootstrap method was used to evaluate the topological stability of the tree with 1,000 replications (14). LL-KF140 was deposited at the Korean Culture Center of Microorganisms under number KCCM11673P.

### Preparation of N^ε^-(Carboxymethyl)lysine and a N^ε^-(Carboxymethyl)lysine-Enriched Casein–Lactose Reaction Product

N^ε^-(Carboxymethyl)lysine was prepared by reacting a milk protein and a carbohydrate. Briefly, sodium casein was added to a 0.25 M lactose solution at a molar ratio of 1:7 (w/w), and the mixture was heated at 140°C for 80 min. The casein and lactose reactant was termed casein-lactose reaction product (CLRP). A commercially synthesized CML was purchased from Santa Cruz Biotechnology.

### Hydrolysis of Advanced Glycation Ends From Casein for Quantification

Proteins from CLRP and LL-KF140 cultured supernatants were extracted using acetone precipitation and were dissolved in 200 μL of 0.01 M HCl. The total protein concentrations in the samples were determined using a bicinchoninic acid assay, and the solutions were diluted to a concentration of 1 mg/mL. Proteases and peptidase were used to hydrolyze the proteins, as reported in previous studies (15, 16). Briefly, the solution was incubated at 37°C for 24 h, followed by the addition of 0.1 mg of pepsin to 1 mL of the protein solution. Next, 250 μL of 2 M Tris-HCl buffer (pH 8.2) and Pronase E (0.1 mg) were sequentially added to the solution, and the solution was incubated at 37°C for 24 h. Finally, the solution was mixed with 1 U of aminopeptidase and 1 U of prolidase, and incubation was continued under the same conditions.

### High-Performance Liquid Chromatography–Electrospray Ionization Mass Spectrometry

All high-performance liquid chromatography–electrospray ionization mass spectrometry (HPLC-ESI-MS) experiments for quantifying AGEs in the samples were performed in positive ion mode using a LCMS-8050 (Shimadzu, Kyoto, Japan) equipped with a Hypercarb porous graphitic carbon column (3 μm, 2.1 mm × 150 mm). The injection volume and the column oven temperature were 4 μL and 40°C, respectively. Two solvents, 0.1% formic acid (FA) in water (solvent A) and 0.1% FA in acetonitrile (solvent B), were used as the mobile phase at a flow rate of 0.2 mL/min. A gradient of the two solvents was set as follows: 0–10.0 min, 0% B; 10.0–15.0 min, 0–20% B; 15.0–20.0 min, 20% B; 20.0–21.5 min, 20–70% B; 21.5–25.0 min, 70% B; 25.0–25.5 min, 70–0% B; 25.5–35.0 min, 0% B. For intact ionization of AGEs, the nebulizing gas, heating gas, and drying gas flow rates were set at 3, 10, and 10 L/min, respectively. In addition, the interface temperature, capillary electrophoresis temperature, and heat block temperature were set to 300, 250, and 400°C, respectively. Quantitative analysis of the samples was performed based on the standard addition method due to the presence of amino acids in the solution.

### Analysis of LL-KF140 Microbial and Enzymatic Reactions With N^ε^-(Carboxymethyl)lysine *in vitro*

LL-KF140 was cultured in De Man, Rogosa, and Sharpe (MRS) broth at 37°C for 24 h. Cultured cells were collected by centrifugation at 2,570 × *g* for 15 min and then washed twice with aseptic phosphate-buffered saline (PBS). The pelleted cells were frozen at –80°C and then lyophilized and stored at –80°C until further use. The viability of lyophilized LL-KF140 cells was determined by colony-forming units (CFU) counts after agar plating.

Total 100 mL of mixture including final CML concentration of 2.49 μg/mL was prepared. In LL-KF140 experiments, 10 mL of a 24-h LL-KF140 culture medium, 10 mL of a prepared CLRP, and 80 mL of M9 broth were mixed. In LL-KF140-free control, 10 mL of a prepared CLRP and 90 mL of M9 broth were mixed. The mixtures were shaken at 100 × *g* at 37°C. The culture and control were incubated in a normal aerobic setup, and the solutions were sampled at different time intervals.

The CML-reducing efficacies of eight enzymes (lactate dehydrogenase, fructose-6-phosphate kinase, β-galactosidase, glucokinase, glutamate dehydrogenase, D-lactate dehydrogenase, glutamate decarboxylase, and UDP-glucose-4-epimerase) that are produced by LL-KF140 were analyzed after incubation of these enzymes with CML for 24 h, as described above.

### Animal Study Design

This study was conducted in accordance with the guidelines of the Institutional Animal Care and Use Committee (IACUC) of the Laboratory Animal Center of the Daegu-Gyeongbuk Medical Innovation Foundation (DGMIF) and approved by the IACUC of the Laboratory Animal Center of the DGMIF (DGMIF-17040502-00). Male Sprague–Dawley rats (6 weeks old) from Orientbio, Inc. (Seongnam, South Korea) were acclimated for 1 week under standard housing conditions at the DGMIF and were provided a standard rodent chow diet and water *ad libitum*. The rats were separated into two experimental groups (LL-KF140-treated and PBS-treated control groups) and orally administered LL-KF140 at 2.0 × 10^8^ CFU/kg body weight in a total volume of 200 μL of PBS once daily for 14 days before CLRP ingestion.

The rats were fasted for at least 14 h prior to beginning the experiment and had free access to tap water. The CLRP was administered at a dose of 10 mg/kg (82.4 ng/kg CML) in a total volume of 10 mL/kg over 0.5 min *via* oral gavage through a feeding tube. Blood samples (0.3 mL) were collected into heparinized tubes *via* the jugular vein at 0 (control), 1, 2, 4, 6, 8, and 24 h after oral administration of the CLRP. The area under the plasma concentration–time curve of CML was calculated.

### Western Blotting

Rat livers were lysed using PRO-PREP™ (iNtRON Biotechnology), and protein concentrations were determined using a protein assay kit (Bio-Rad). Proteins were separated by sodium dodecyl sulfate–polyacrylamide gel electrophoresis and then transferred onto polyvinylidene difluoride membranes. The membranes were incubated overnight at 4°C with primary antibodies against CML, receptor for advanced glycation end products (RAGE), and β-actin (Abcam), followed by incubation with the rabbit secondary antibody (Thermo Fisher Scientific, Waltham, MA, United States), and the protein bands were visualized using a ChemiDoc XRS + imaging system (Bio-Rad, Hercules, CA, United States).

### Human Participants and the Clinical Trial

The clinical trial was performed at the Clinical Trial Convergence Commercialization Team at the Daegu Technopark (Daegu, South Korea). The inclusion criteria were as follows: (a) men and women aged 25–45 years; (b) participants with a serum CML level of 2.1 ± 0.4 μg/mL; and (c) participants who understood the objectives of the study and agreed to abide by the required rules during the study. Patients with diabetes and those with vascular disease, heart disease, or any previous medical history of these conditions within the last 12 months were excluded. In addition, patients who had recently taken probiotics that could interfere with the efficacy evaluation were excluded. The protocol was approved by the Institutional Review Board (IRB) of the Daegu Haany University Korean Medicine Hospital (Daegu, South Korea) (IRB approval number: DHUMC-D-17029).

In total, 12 subjects passed the selection/exclusion screening and were enrolled into the study; however, data from 11 subjects were analyzed (1 subject dropped out following a compliance issue).

Subjects were hospitalized for 2-night/3-day (visit 2) within 20 days from the selected inspection date (visit 1) and were provided the test product on the discharge date. LL-KF140 at 2.0 × 10^9^ CFU/1.5 g was ingested by the participants for 26 days, one packet each morning starting from the morning after discharge. The study protocol was terminated in the second 2-night/3-day hospitalization (visit 3) and completing and submitting all scheduled tests and specimens. On day 2, the participants were asked to consume 40 g of a parmesan cheese, containing 10 mg/g CML, within 30 min, and after completion of the breakfast, 6 mL of venous serum was collected at 1, 2, 4, 6, 8, 12, and 24 h using a serum separate tube. Fecal samples were collected once during each hospital visit and stored at –80°C.

### Determination of Serum Levels of N^ε^ -(Carboxymethyl)lysine and Biomarkers

Both rat and human serums were immediately placed on ice, allowed to sit for 10–15 min to facilitate clotting, and centrifuged at 1,500 rpm for 30 min. The serum samples were separated, aliquoted, and stored at –80°C.

N^ε^-(Carboxymethyl)lysine levels were measured in the serum using a CML enzyme-linked immunosorbent assay (ELISA) kit (CircuLex). Samples of rat and human sera were collected according to the manufacturer’s recommendations, and CML levels were then determined by the ELISA in triplicate, with the absorbance measured at a wavelength of 450 nm using a spectrophotometer. The CML concentration was then calculated using a calibration curve created with known concentrations of CML–human serum albumin (0.109–14.000 μg/mL). Serum samples were also analyzed for hemoglobin A1c (HbA1c), total cholesterol (TC), triglycerides (TG), low-density lipoprotein cholesterol (LDL-C), high-density lipoprotein cholesterol (HDL-C), aspartate aminotransferase (AST), and alanine aminotransferase (ALT) using commercially available kits (Abcam).

### Stool 16S rRNA and Metagenomics Sequencing

Fresh stool samples were obtained from the 11 healthy participants and frozen at –80°C in sterile containers. Stool samples from each individual were used for the microbial community analysis. DNA was extracted, and the V3–V4 hypervariable region of the 16S rRNA gene was amplified using a MiSeq instrument (Illumina, San Diego, CA, United States) at Macrogen (Seoul, South Korea).

Operational taxonomic units (OTUs) for each sample were analyzed using CD-HIT and clustered with a threshold of 97% sequence homology (17). The taxonomic classification of OTUs was carried out by QIIME-UCLUST using the dataset from the Ribosomal Database Project (18). The Wilcoxon signed-rank test was applied to compare differential values of taxonomic assignments before and after LL-KF140 ingestion, and the data were visualized using the R software.^3^

### Statistical Analysis

Data are expressed as the mean ± standard deviation (SD; *in vitro*) or standard error of the mean (SEM; *in vivo*), unless otherwise indicated, and were analyzed using the GraphPad Prism software (GraphPad Software, San Diego, CA, United States). Statistical analysis included the Student’s *t-*test and one-way analysis of variance (ANOVA) with Tukey’s test, when appropriate. Statistical significance was set at *p* < 0.05.

## Results

### Identification of LL-KF140

Various potential probiotics were isolated from kimchi, and their ability to reduce CML levels was investigated (data not shown). The isolated KF140 strain, with a high CML-reducing activity, was gram-negative and catalase-negative and grew as single cells, with the formation of white colonies on MRS agar plates. Strain KF140 was able to utilize the following 20 carbohydrates: ribose, xylose, galactose, glucose, fructose, mannose, mannitol, *N*-acetylglucosamine, amygdalin, arbutin, esculin, salicin, cellobiose, maltose, lactose, sucrose, trehalose, starch, β-gentiobiose, and gluconate (Supplementary Table 1). In addition, strain KF140 did not show antibiotic resistance or hemolytic activity (Supplementary Table 2).

**FIGURE 1:**
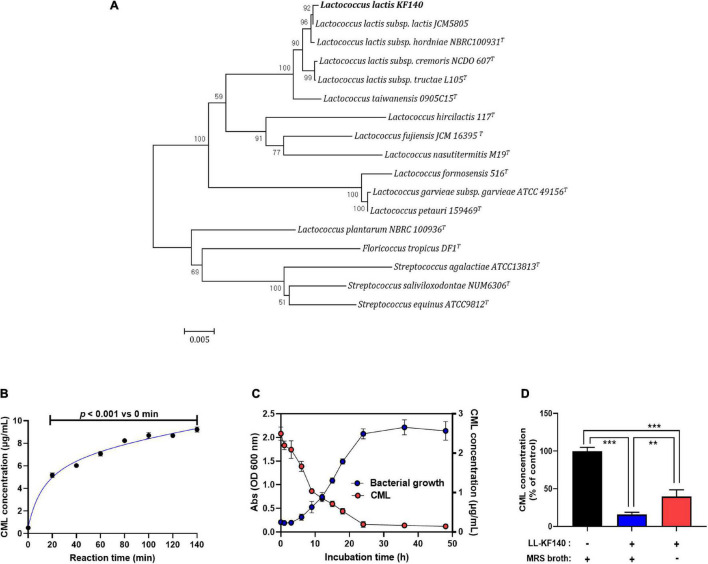
Isolation of *Lactococcus lactis* KF140 (LL-KF140) and its reduction of *N*^ε^ -(Carboxymethyl)lysine (CML) concentration *in vitro* test. **(A)** Neighbor-joining phylogenetic tree based on 16S rRNA gene sequences, showing the positions of *L. lactis* KF140 and other closely related lactic acid bacteria species. Bootstrap values (expressed as percentages of 1,000 replications) are shown at branching points. **(B)** Formation of CML following the reaction of casein and lactose at 140°C for different times. **(C)** Bacterial growth curve and the effect of LL-KF140 on the CML concentration *in vitro*. **(D)** Reduction of CML levels by LL-KF140 with or without MRS growth medium. Data are presented as the mean ± SD (*n* = 3). **p < 0.01, ***p < 0.001 (one-way ANOVA, followed by Tukey’s test).

Phylogenetic relationships of the novel bacterial isolate were analyzed using 16S rRNA gene sequencing. In the constructed phylogenetic trees, strain KF140 was clustered with *L. lactis* strains and well-matched reference strains (Figure 1A). Thus, KF140 was identified as a strain of *L. lactis* (98% sequence homology).

### *In vitro* Reduction of the N^ε^ -(Carboxymethyl)lysine Level by LL-KF140

After a milk protein (casein) and lactose were heated for 80 min at 140°C, the CML content increased by 8 μg/mL (Figure 1B). Upon incubation of LL-KF140 with CLRP, the CML concentration decreased as the bacterial growth progressed and reached a minimum when the bacterial growth reached the stationary phase (Figure 1C). Based on this observation, the CLRP (2.7 mg/mL) was heated at 140°C for 80 min and used to assess the ability of LL-KF140 to reduce the level of CML. LL-KF140 decreased the level of CML by 60% in non-nutritive M9 culture medium and by 80% when MRS broth (nutritive) was used (Figure 1D).

### *In vivo* Inhibition of N^ε^ -(Carboxymethyl)lysine Absorption by LL-KF140

**FIGURE 2:**
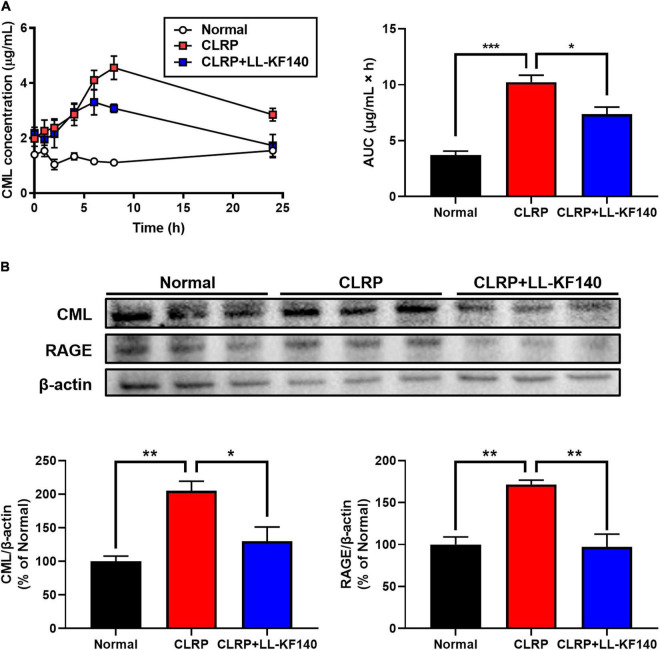
Effect of oral administration of *Lactococcus lactis* KF140 (LL-KF140) on *N*^ε^ -(Carboxymethyl)lysine (CML) levels in the serum and liver tissues of rats after 10 mg/kg CML administration in a casein-lactose reaction product (CLRP). **(A)** Serum CML levels in rats with and without LL-KF140 administration. **(B)** Levels of CML and the receptor for advanced glycation end products (RAGE) in rat liver tissues. Data are presented as the mean ± SEM (*n* = 3). *p < 0.05, **p < 0.01, ***p < 0.001 (one-way ANOVA, followed by Tukey’s test).

A rat model was used to evaluate whether LL-KF140 could reduce CML absorption *in vivo*. The experiment involved the administration of LL-KF140 to rats at a dose of 2.0 × 10^8^ CFU/kg for 14 days, followed by the administration of 10 mg/kg CLRP (82.4 ng/kg CML). In the CLRP alone treatment group, the serum levels of CML peaked at 8 h and then decreased at 24 h after CLRP administration (*p* < 0.05). However, coadministration of LL-KF140 resulted in a 27% lower CML level than that in the CLRP alone treatment group 24 h post-administration (*p* < 0.05; Figure 2A). In addition, the measurement of blood levels of AST, ALT, TC, TG, LDL-C, and HDL-C 24 h after CLRP administration showed that these indicators were unaffected by LL-KF140 administration (Supplementary Table 3).

Next, the accumulation of CML and the expression of the RAGE were analyzed in rat liver tissues (Figure 2B). After 24 h, CLRP significantly increased the levels of CML and RAGE; however, LL-KF140 strongly inhibited these CLRP-mediated increases (*p* < 0.05 and *p* < 0.01, respectively). These results suggest that LL-KF140 reduces the absorption of CML and prevents the accumulation of AGEs in liver tissue.

### Effect of LL-KF140 on Serum N^ε^ -(Carboxymethyl)lysine Levels in Humans

**FIGURE 3:**
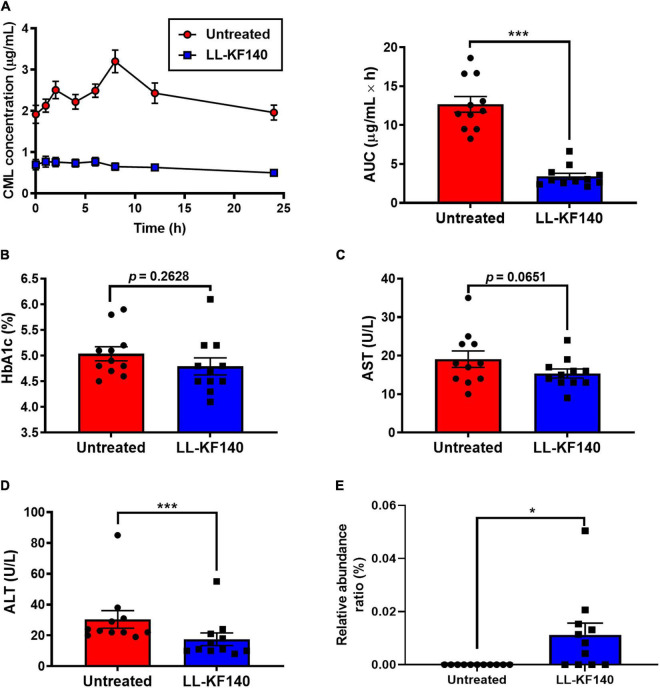
Effect of ingesting *Lactococcus lactis* KF140 (LL-KF140) on serum levels of *N*^ε^ -(Carboxymethyl)lysine (CML) in human subjects. **(A)** Serum CML levels after intake of a CML-enriched parmesan cheese, before and 26 days after LL-KF140 administration. Serum concentrations of **(B)** HbA1c, **(C)** AST, and **(D)** ALT. **(E)** Relative abundances of *L. lactis* in stool samples of human subjects before and after LL-KF140 administration. Data are presented as the mean ± SEM (*n* = 11). *p < 0.05, ***p < 0.001 (paired two-tailed Student’s *t*-test).

To evaluate whether LL-KF140 could inhibit the absorption of CML in humans, blood tests were performed after LL-KF140 intake for 26 days, followed by the ingestion of a parmesan cheese. Figure 3A depicts that the intake of CML contained parmesan cheese gradually elevated CML levels until 8 h, and then the levels returned to the initial values within 24 h. However, after LL-KF140 consumption for 26 days resulted in significantly lower CML levels (*p* < 0.001) than those in the untreated group over the period of observation (Figure 3A).

The HbA1c and AST levels were not significantly changed by LL-KF140 administration (from 5.0 to 4.7% and from 19.1 to 15.4 U/L, respectively) (Figures 3B,C). However, the levels of ALT (*p* < 0.001) and LDL-C (*p* < 0.01) were significantly reduced upon LL-KF140 intake, from 30.5 to 17.5 and from 115.3 to 95.5 U/L, respectively (Figure 3D and Supplementary Table 4).

### Changes in the Fecal Microbiota

To assess whether orally administered LL-KF140 affects the gut microbiota composition, the presence of LL-KF140 was determined in fecal samples of the human participants. LL-KF140 was not detected in stool samples before the ingestion; however, 26 days after treatment, LL-KF140 was observed in the assessed stool samples (Figure 3E). Moreover, the administration of LL-KF140 changed the abundances of 11 gut microbes at the species level (Supplementary Table 5). These results indicate that prolonged consumption of LLF-KF140 lower blood CML level after the consumption of CML-reach food.

### Effect of LL-KF140 Enzyme Activity on N^ε^ -(Carboxymethyl)lysine Levels

**FIGURE 4:**
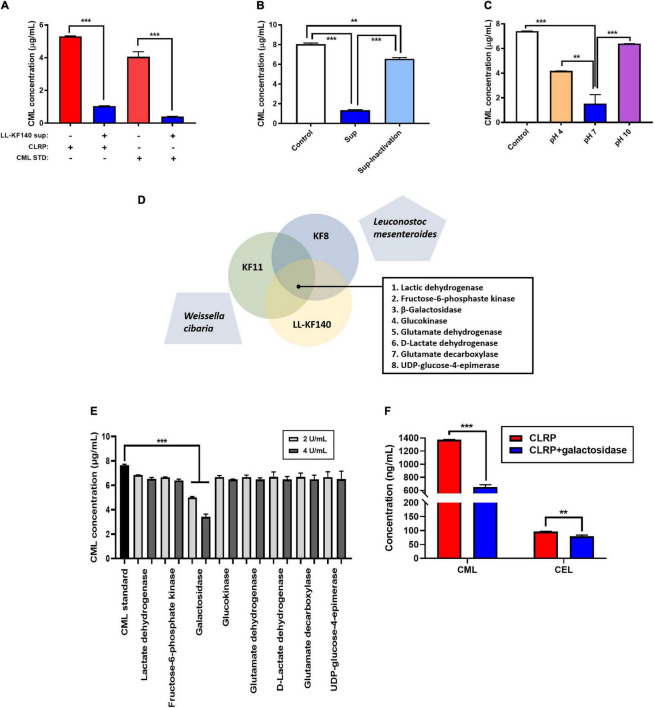
*In vitro* effects of the *Lactococcus lactis* KF140 (LL-KF140) culture supernatant and related enzymes on *N*^ε^ -(Carboxymethyl)lysine (CML) levels. **(A)** CML levels in the casein-lactose reaction product (CLRP) and synthetic CML after incubation with the LL-KF140 supernatant. **(B)** Effects of the LL-KF140 supernatant, with and without heat inactivation, on CML levels. **(C)** Effects of the LL-KF140 supernatant on CML levels under different pH conditions. **(D)** Selected enzymes that could potentially reduce the levels of CML. **(E)** Effects of LL-KF140-producing enzymes on CML levels. **(F)** Effects of β-galactosidase on CML and *N*^ε^ -(carboxyethyl)lysine (CEL) levels. Data are presented as the mean ± SD (*n* = 3). **p < 0.01, ***p < 0.001 (one-way ANOVA, followed by Tukey’s test, or a paired two-tailed Student’s *t*-test).

To elucidate the mechanism of action of LL-KF140 in lowering CML levels, we determined whether the reduction in CML levels by LL-KF140 could be due to enzymatic activity. As shown in Figure 4A, the levels of CML were significantly reduced (*p* < 0.001) after the incubation of either the CLRP or synthetic CML with an LL-KF140 culture supernatant. However, heat inactivation of the LL-KF140 culture medium significantly suppressed its CML-reducing efficacy (*p* < 0.001; Figure 4B). Moreover, the effect of the LL-KF140 culture supernatant in reducing CML levels was significantly weakened under acidic and alkaline pH conditions (*p* < 0.01 and *p* < 0.001, respectively; Figure 4C). We hypothesized that the CML-lowering effect of LL-KF140 was associated with an enzyme activity. Eight enzymes (Figure 4D) which were commonly secreted by three CML-reducible bacteria strains, *Lacticaseibacillus paracasei* KF00816, *Lactiplantibacillus pentosus* KF8, and *Bacillus subtilis* KF11, but not by *Leuconostoc mesenteroides* and *Weissella cibaria*, which did not reduce CML levels (data not shown). Among the eight enzymes, only β-galactosidase (2 and 4 U/mL) significantly reduced the levels of CML (by 35 and 55%, respectively) compared with that in the synthetic CML group (Figure 4E). We further confirmed, using HPLC-ESI-MS, that β-galactosidase treatment significantly reduced the contents of CML and *N*^ε^ -(carboxyethyl)lysine (CEL) (*p* < 0.001 and *p* < 0.01, respectively; Figure 4F).

## Discussion

It has been reported that different kinds of beneficial commensal lactobacilli are part of the normal intestinal microbiota and play an important role in human health by preventing intestinal infections, lowering cholesterol levels, stimulating the immune system, and reducing the risk of colon cancer (19). Probiotic bacteria produce lactic acid and other organic acids, reduce the pH of the microenvironment, and tend to prevent the growth of gram-negative bacteria that contain lipopolysaccharides (20). These commensal bacteria also produce antimicrobial compounds, such as bacteriocins, that can regulate the gut microbiota (21). Another function that is associated with intestinal microbes is detoxification. Many studies have reported a removal of heavy metals and toxic substances by probiotic microorganisms (22, 23). More studies should be performed to evaluate the action of dietary toxic substances and the possible role of probiotic bacteria in their detoxification.

*Lactococcus lactis* strains have been used to ferment foods for centuries and obtained a GRAS status from the Food and Drug Administration. In this study, LL-KF140, which did not show resistance to antibiotics, was isolated from kimchi. In addition, we prepared a dietary CML, termed the CLRP, at a concentration of 2.7 mg/mL through the reaction of a milk protein and a carbohydrate. As it grew, LL-KF140 reduced the CML content in the CLRP, not only in a nutritive medium but also in a non-nutritive medium. We demonstrated the efficacy of LL-KF140 in reducing CML levels in *in vitro* experiments and also investigated the toxicokinetics of CML after LL-KF140 administration *in vivo*.

An *L. lactis* strain has been reported to exert detoxification effects against xenobiotics (23, 24). In this study, to determine the effect of LL-KF140 on the toxicokinetics of CML, LL-KF140 was administered to rats at a dose of 2.0 × 10^8^ CFU/kg for 14 days, followed by the administration of 10 mg/kg CLRP (82.4 ng CML/kg). In our CML-induced rat model, administration of LL-KF140 inhibited the accumulation of CML in the blood and liver and the expression of the RAGE in the liver but had no effect on serum AST, ALT, and lipid levels. Excessive consumption of AGEs by animals can cause the onset of liver inflammation, even in the absence of steatosis, which is considered to be due to the absorption of AGEs by liver tissue (25). In addition, increases in AGE levels and AGE–RAGE interactions increase the production of reactive oxygen species, expression of inflammatory markers, and activation of the NF-κB signaling pathway (26). Our findings suggest that LL-KF140 intake can potentially prevent the inflammatory response caused by CML consumption by suppressing CML accumulation and RAGE expression in liver tissues. Numerous studies, most of which have been conducted using high-fat diet-induced animal models and long-term intake of probiotics, have reported lowering effects of probiotics on the levels of cholesterol and liver damage parameters (27, 28). Similarly, in our human trial, after LL-KF140 intake for 26 days, followed by the ingestion of 40 g of a parmesan cheese containing 10 mg/g CML, serum CML, ALT, and LDL-C levels did not significantly increase; however, the levels of serum HbA1c, AST, TC, and TG were not affected. Therefore, additional studies are needed to determine the duration of LL-KF140 intake that would yield the most efficient results for serum biomarkers in animals and humans after CML administration. Furthermore, HbA1c is an index for evaluating chronic glycemia over approximately 3 months (29), and our findings suggest that HbA1c is not affected by short-term administration of LL-KF140.

In a human study, consumption of LL-KF140 for 26 days showed an efficacy in reducing serum CML level and determined to presence in feces through metagenomics sequencing. However, no significant difference was found in the correlation analysis between the reduction in CML and LL-KF140 administration. Incubation of the synthetic CML and CLRP with the supernatant of LL-KF140 resulted in the reduction of CML levels. However, heat treatment of the supernatant significantly weakened this effect, and the optimum efficacy was observed at pH 7. These data indicated that an enzyme activity was involved in the CML-reducing activity of LL-KF140, and the evaluation of eight *L. lactis* producing enzymes showed that β-galactosidase had the strongest effect. Furthermore, β-galactosidase significantly reduced the levels of CML and CEL during incubation with the CLRP. Similar to CML, CEL is also formed in both food and biological systems through the Maillard reaction, which combines reducing sugars and lysine to form Amadori products, and CEL also induces an inflammatory response *via* RAGE signaling (30). Galactosidase has great potential in both medical and biotechnological applications and great utility in the food industry (31, 32). Based on our findings, we suggest a broader use of probiotics, including LL-KF140, in medical and biotechnological applications because of their CML-reducing properties, which are associated with β-galactosidase, although further investigations are required in larger study cohorts using a blinded, placebo-controlled design. Thus, it is necessary to present the correlation between bacterial diversity, galactosidase activity, and changes in blood CML level through additional animal experiments in the future.

## Conclusion

The main finding of the study was that β-galactosidase, which is produced by LL-KF140, was able to reduce the levels of two AGEs. This study is the first to demonstrate that LL-KF140 reduces CML levels or absorption owing to the activity of β-galactosidase. In addition, prolonged consumption of LL-KF140 is associated with the lower level of CML in blood after the consumption of CML-reach food and that the presence in the gut microbiota. However, further studies are needed to elucidate the mechanisms of action of galactosidase and establish the dosage and duration of LL-KF140 intake.

## Data Availability Statement

The datasets presented in this study can be found in online repositories. The names of the repository/repositories and accession number(s) can be found below:

https://figshare.com/, 19555408

https://www.ncbi.nlm.nih.gov/genbank/, PRJNA699930.

## Ethics Statement

The studies involving human participants were reviewed and approved by Institutional Review Board (IRB) of the Daegu Haany University Korean Medicine Hospital (Daegu, South Korea) (IRB approval number: DHUMC-D-17029). The patients/participants provided their written informed consent to participate in this study. The animal study was reviewed and approved by Institutional Animal Care and Use Committee (IACUC) of the Laboratory Animal Center of the Daegu-Gyeongbuk Medical Innovation Foundation (DGMIF) and approved by the IACUC of the Laboratory Animal Center of the DGMIF (DGMIF-17040502-00). Written informed consent was obtained from the individual(s) for the publication of any potentially identifiable images or data included in this article.

## Author Contributions

H-YP, HL, and YK provided major input into the conceptual development of the studies, analyzed the collected data, wrote the manuscript, and supervised all of the investigations. H-YP, M-JO, SH, and ED managed and treated the enrolled patients and collected the data. S-YL, HL, JH, and MP conducted formal analysis. K-WL and M-HN reviewed the manuscript. All authors contributed to the article and approved the submitted version.

## Conflict of Interest

MP was employed in MetaCenTherapeutics. The remaining authors declare that the research was conducted in the absence of any commercial or financial relationships that could be construed as a potential conflict of interest.

## Publisher’s Note

All claims expressed in this article are solely those of the authors and do not necessarily represent those of their affiliated organizations, or those of the publisher, the editors and the reviewers. Any product that may be evaluated in this article, or claim that may be made by its manufacturer, is not guaranteed or endorsed by the publisher.

## Footnotes

1
https://www.ezbiocloud.net/

2
https://www.genome.jp/tools-bin/clustalw

3
www.r-project.org
