# Supplementary material for: Lactococcus lactis KF140 Reduces Dietary Absorption of Nε - (Carboxymethyl)lysine in Rats and Humans via β-Galactosidase Activity
Source: Front Nutr. 2022 Jun 24;9:916262. doi: 10.3389/fnut.2022.916262 (PMC9263842; doi:10.3389/fnut.2022.916262)
Supplement: Supplementary file 1 [file Data_Sheet_1.docx]

**SUPPLEMENTARY MATERIAL**

***Lactococcus lactis* KF140 reduces dietary absorption of *N*^ε^-(carboxymethyl)lysine in rats and humans via β-galactosidase activity**

Ho-Young Park^1,a^, Hye-Bin Lee^1,a^, So-Young Lee^1^, Mi-Jin Oh^1^, Sang Keun Ha^1^, Eunju Do^2^, Hyun Hee L. Lee^2^, Jinyoung Hur^1^, Kwang-Won Lee^3^, Mi-Hyun Nam^4^, Myoung Gyu Park^5^, and Yoonsook Kim^1,^*

^1^*Food Functionality Research Division, Korea Food Research Institute, Jeollabuk-do 55365, Republic of Korea*; ^2^*Clinical Trial Convergence Commercialization Team, Daegu Technopark, Daegu 42158, Republic of Korea*; ^3^*Department of Biotechnology, College of Life Sciences and Biotechnology, Korea University, Seoul 02841, Republic of Korea*; ^4^*Sue Anschutz-Rodgers Eye Center and Department of Ophthalmology, University of Colorado, Aurora, CO 80045, U.S.A.*; ^5^*MetaCenTherapeutics, Gyeonggi-do 16229, Republic of Korea*

^a^ These authors contributed equally to this work.

*Corresponding author: Yoonsook Kim

E-mail address: kimyus@kfri.re.kr, kys7809281@gmail.com

Address: 245 Nongsaengmyeong-ro, Iseo-myen, Jeollabuk-do 55365, Republic of Korea

Telephone: +82-63-219-9281; Fax: +82-63-219-9876

**Competing Interests:** The authors declare no competing interests.

**Tables**

**Table S1. Biochemical characteristics of *Lactococcus lactis* KF140 using API 50 CHL.**

| Carbohydrate | Utilized | Carbohydrate | Utilized |
| --- | --- | --- | --- |
| Glycerol | - | Salicine | + |
| Erythritol | - | Cellobiose | + |
| D-Arabinose | - | Maltose | + |
| L-Arabinose | - | Lactose | + |
| Ribose | + | Melibiose | - |
| D-Xylose | + | Saccharose(Sucrose) | + |
| L-Xylose | - | Trehalose | + |
| Adonitol | - | Inuline | - |
| β-Methyl-xyloside | - | Melezitose | - |
| Galactose | + | D-Raffinose | - |
| D-Glucose | + | Amidon | + |
| D-Fructose | + | Glycogene | - |
| D-Mannose | + | Xylitol | - |
| L-Sorbose | - | β-gentiobiose | + |
| Rhamnose | - | D-Turanose | - |
| Dulcitol | - | D-lyxose | - |
| Inositol | - | D-Tagatose | - |
| Mannitol | + | D-Fucose | - |
| Sorbitol | - | L-Fucose | - |
| β-Methyl-D-manoside | - | D-Arabitol | - |
| α-Methyl-D-glucoside | - | L-Arabitol | - |
| N-Acetyl glucosamine | + | Gluconate | + |
| Amygdaline | + | 2-Ceto-gluconate | - |
| Arbutine | + | 5-Ceto-gluconate | - |
| Exculine | + |  |  |

**Table S2. Antibiotic susceptibility of the *Lactococcus lactis* KF140 (LL-KF140).**

| Antibiotic | LL-KF140 (µg/ml) | |
| --- | --- | --- |
|  | EFSA (2012) ^a^ | IST+MRS sensititre ^b^ |
| Penicillin |  | 0.25 |
| Ampicillin | 2 | 0.25 |
| Oxacillin+2%NaCl |  | 1 |
| Cefoxitin |  | 6 < |
| Vancomycin | 4 | 0.5 |
| Chloramphenicol | 8 | 4 |
| Gentamicin | 32 | 4 |
| Streptomycin | 32 | 16 |
| Quinupristin/dalfopristin |  | 1 |
| Rifampin |  | 4< |
| Tetracycline | 4 | 2 |
| Erythromycin | 1 | 0.25 |
| Tigecycline |  | 0.06 |
| Clindamycin | 1 | 0.5 |
| Linezolid |  | 2 |
| Trimethoprim/sulfamethoxazole |  | 4/76 < |
| Levofloxacin |  | 2 |
| Moxifloxacin |  | 2 |
| Ciprofloxacine |  | 2 < |
| Nitrofurantoin |  | 32 |
| Daptomycin |  | 0.5 |

^a^ Microbiological breakpoints are the values determined by EFSA above which a *Lactococcus lactis* is considered resistant (EFSA, 2012)

^b^ IST+MRS, a mixed formulation of Iso-Sensitest broth (90%) and deMan-Rogosa-Sharpe broth (10%)

**Table S3. Effects of the *Lactococcus lactis* KF140 (LL-KF140) on aspartate transaminase (AST), alanine transaminase (ALT), total cholesterol, low-density lipoprotein (LDL)-cholesterol, high-density lipoprotein (HDL)-cholesterol, and triglyceride levels in serum after 24 h of 10 mg/kg casein-lactose reactant (CLR) administered rats.**

|  | AST  (U/L) | ALT  (U/L) | Total cholesterol (mg/dL) | LDL-cholesterol  (mg/dL) | HDL-cholesterol  (mg/dL) | Triglyceride  (mg/dL) |
| --- | --- | --- | --- | --- | --- | --- |
| CLR | 66.3 ± 2.4 ^a^ | 25.0 ± 3.2 | 44.3 ± 11.4 | 8.3 ± 0.7 | 39.7 ± 0.9 | 42.7 ± 1.8 |
| CLR+  LL-KF140 | 62.0 ± 3.1 | 23.3 ± 1.3 | 40.3 ± 5.9 | 7.3 ± 0.9 | 40.7 ± 4.7 | 40.3 ± 2.0 |
| *p*-value | NS ^b^ | NS | NS | NS | NS | NS |

^a^ Each value provided is the mean ± SEM for 3 rats.

^b^ NS: not significant.

**Table S4. Effects of the *Lactococcus lactis* KF140 (LL-KF140) on total cholesterol, low-density lipoprotein (LDL)-cholesterol, high-density lipoprotein (HDL)-cholesterol, and triglyceride levels in serum of human who administered with 2.0 × 10^9^ CFU LL-KF140/day for 26 days**

|  |  | Total cholesterol (mg/dL) | LDL cholesterol  (mg/dL) | HDL cholesterol  (mg/dL) | Triglyceride  (mg/dL) |
| --- | --- | --- | --- | --- | --- |
| LL-KF140 intake | Before | 162.5 ± 6.9 ^a^ | 115.3 ± 5.2 | 48.2 ± 3.4 | 86.0 ± 11.8 |
|  | After | 157.4 ± 5.6 | 95.5 ± 4.7 | 49.0 ± 3.5 | 107.4 ± 6.4 |
|  | p-value | NS ^b^ | *p* < 0.01 | NS | NS |

^a^ Each value provided is the mean ± SEM for 11 subjects.

^b^ NS: not significant.

**Table S5. Significant taxonomic changes in stool microbiota after 26 days administration of *Lactococcus lactis* KF140 at a dose of 2.0 × 10^9^ CFU/day.**

| Species | Before (%) | After (%) | p-value ^b^ |
| --- | --- | --- | --- |
| *Fusicatenibacter saccharivorans* | 0.207 ± 0.362 | 0.575 ± 0.934 | 0.002 |
| *Ruminococcus callidus* | 0.204 ± 0.391 | 1.096 ± 2.114 | 0.002 |
| *Romboutsia timonensis* | 0.011 ± 0.010 | 0.057 ± 0.061 | 0.014 |
| *[Eubacterium] rectale* | 0.956 ± 1.091 | 3.485 ± 3.560 | 0.020 |
| *Flavonifractor plautii* | 0.075 ± 0.062 | 0.018 ±0.022 | 0.021 |
| *Lactococcus lactis* | 0.000 ± 0.000 | 0.012 ±0.015 | 0.022 |
| *Ruminococcus bromii* | 0.046 ± 0.070 | 0.196 ± 0.306 | 0.022 |
| *Ruminococcus champanellensis* | 1.803 ± 2.726 | 0.624 ± 0.822 | 0.024 |
| *[Clostridium] leptum* | 0.011 ± 0.009 | 0.023 ± 0.018 | 0.030 |
| *Dorea longicatena* | 0.044 ± 0.057 | 0.015 ± 0.026 | 0.033 |
| *Dorea formicigenerans* | 0.050 ± 0.059 | 0.192 ± 0.346 | 0.037 |

^a^ Each value provided is the mean ± SD for 11 subjects.

^b^ Paired sample Wilcoxon signed-rank test was used to analyze variations between before and after ingestion.
